# Unique loop-structured CD19/CD22 bispecific CAR-T-cell therapy for patients with relapsed/refractory diffuse large B-cell lymphoma: an observational study

**DOI:** 10.1093/abt/tbaf027

**Published:** 2025-11-20

**Authors:** Shuhong Li, Liqiong Liu, Zelin Liu, Jianjiang Li, Huanhuan Zhou, Nan Zhong, Yuan Ye, Lijun Zhao, Xiao Liang, Yuanyuan Shi, Yu J Cao, Zhi Guo

**Affiliations:** State Key Laboratory of Chemical Oncogenomics, Shenzhen Key Laboratory of Chemical Genomics, Peking University Shenzhen Graduate School, Shenzhen 518055, China; Department of Hematology, Affiliated Nanshan Hospital of Shenzhen University, Shenzhen 518052, China; Department of Hematology, Affiliated Nanshan Hospital of Shenzhen University, Shenzhen 518052, China; State Key Laboratory of Chemical Oncogenomics, Shenzhen Key Laboratory of Chemical Genomics, Peking University Shenzhen Graduate School, Shenzhen 518055, China; Department of Hematology, Affiliated Nanshan Hospital of Shenzhen University, Shenzhen 518052, China; Department of Hematology, Affiliated Nanshan Hospital of Shenzhen University, Shenzhen 518052, China; Department of Hematology, Affiliated Nanshan Hospital of Shenzhen University, Shenzhen 518052, China; Shenzhen Cell Valley Biomedical Co., Ltd, Shenzhen 518118, China; National Engineering Research Center for Foundational Technologies for CGT Industry, Shenzhen 518055, China; Shenzhen Cell Valley Biomedical Co., Ltd, Shenzhen 518118, China; State Key Laboratory of Chemical Oncogenomics, Shenzhen Key Laboratory of Chemical Genomics, Peking University Shenzhen Graduate School, Shenzhen 518055, China; Shenzhen Bay Laboratory, Institute of Chemical Biology, Shenzhen 518132, China; Department of Hematology, Affiliated Nanshan Hospital of Shenzhen University, Shenzhen 518052, China

**Keywords:** bispecific CAR-T cells, CD19, CD22, DLBCL, antigen escape

## Abstract

**Background:**

Although CD19 and CD22 chimeric antigen receptor (CAR-T) cell therapies have demonstrated encouraging clinical responses in patients with B-cell lymphoma, over 50% of patients ultimately experience disease progression due to frequent antigen escape. The development of CD19/CD22 dual-target CAR-T cells holds promise for overcoming this limitation; however, their clinical application is currently challenging because of insufficient targeting of CD22.

**Methods:**

In this study, we engineered CD19/CD22 BS Loop CAR-T cells with an enhanced targeting efficacy for CD22 and assessed their safety and effectiveness in patients with relapsed/refractory diffuse large B-cell lymphoma.

**Results:**

Among the five patients who received CD19/CD22 bispecific Loop CAR-T-cell therapy (1.6 × 10^6^/kg) from December 2023 to May 2024, four patients (80%) achieved complete remission (CR), and one patient (20%) maintained a stable disease status 1 month after infusion. The expansion of the CD19/CD22 Beta-stranded (BS) Loop CAR-T cells was effective *in vivo* and detectable in the peripheral blood. All patients experienced only Grade 0–1 cytokine release syndrome without any observed neurotoxicity. With the follow-up extended to May 2025 (lasting for at least 1 year), three patients experienced disease progression and eventually died, while the remaining two patients remained in CR.

**Conclusions:**

CD19/CD22 BS Loop CAR-T-cell therapy exhibits potent antilymphoma activity while addressing the challenges associated with designing CAR-T cells that are equally potent against two antigens. This treatment may represent a safe and effective unique immunotherapeutic strategy for lymphoma.

## Teaser

**Statement of Significance**
 

- A unique loop structure-based CD19/CD22 bispecific chimeric antigen receptor (CAR) increases dual-targeting efficacy.
- The CD19/CD22 BS Loop CAR addresses the challenge of insufficient CD22 targeting.
- CD19/CD22 BS Loop CAR-T cells were well tolerated and effective for r/r DLBCL.
- CD19/CD22 BS Loop CAR-T cell therapy represents a promising strategy for lymphoma.

## Graphical Abstract

**Graphical Abstract:**
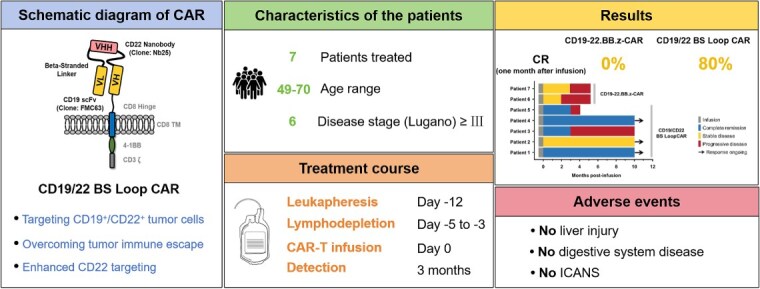

## Introduction

Chimeric antigen receptor T-cell (CAR-T) therapy has driven a paradigm shift in the treatment of relapsed/refractory diffuse large B-cell lymphoma (r/r DLBCL), demonstrating remarkable antitumor effects [1, 2]. Despite the clinical benefits of CAR-T-cell therapy, 60%–70% of r/r DLBCL patients fail to achieve sustained remission after receiving CD19 CAR-T-cell therapy [3]. High recurrence rates due to antigen escape and CAR-T-cell exhaustion limit the efficacy and widespread clinical application of CAR-T-cell therapy in patients with r/r DLBCL [4].

Dual- or multiantigen targeting of CAR-T cells can reduce antigen-negative relapse and prolong the duration of remission [5, 6]. CD22 is a sialic acid-binding adhesion molecule with high expression in most B-cell malignancies [7]. Several studies have indicated that dual targeting of CD19 and CD22 is a rational design approach [5, 8, 9]. Dual antigen targeting could be achieved through several methods, including coadministration of single-target products, cotransduction of two single-target CAR vectors, bispecific receptors containing two independent receptors, and tandem- or loop-structured dual-target CARs [10, 11]. To date, there is still a lack of sufficient data to determine the most effective dual-targeting approach for achieving optimal clinical outcomes.

Clinical cases have shown that antigen escape still occurs even with sequential and cocktail therapy involving CD22 and CD19 single-target CAR-T cells, which indicates the limitations of these dual-targeting approaches [7, 12, 13]. Recently, various CAR constructs for CD19 and CD22 dual targeting have been designed, effectively overcoming tumor heterogeneity and reducing the recurrence rates [5, 8, 14–16]. The CAR structure that binds to the appropriate domains of both the CD19 and CD22 antigens simultaneously may be a key factor in triggering robust CAR-T-cell activity [15, 17–19]. However, almost all clinically studied CD19/CD22 dual-specific CAR-T cells currently pose the problem of suboptimal single-response activity, especially for the CD22 target [9, 15, 17–19]. As we reported previously [9], a unique loop-structured CD19/CD22 bispecific CAR (CD19/CD22 BS Loop CAR) ensured the response activity of CD19 and CD22 dual targeting, which greatly compensated for this drawback.

In a clinical trial of adults with r/r DLBCL, we tested unique loop-structured CD19/CD22 BS Loop CAR-T cells and demonstrated their outstanding clinical efficacy (80% CR) and well-tolerated toxicity in r/r DLBCL patients. Although the clinical sample size was limited, the preliminary results demonstrated that the CD19/CD22 BS Loop CAR-T cells exhibited promising efficacy and safety in treating patients with r/r DLBCL, particularly showing significant advantages in CD22 targeting. More importantly, these findings further validate our unique insights into the optimization of dual-targeting CAR structures, providing critical theoretical support for the future design of more effective CAR-T cell therapies.

## Materials and methods

### Patient information

We assessed the safety and feasibility of CD19/CD22 BS Loop CAR-T-cell therapy in adult patients with r/r DLBCL at the Nanshan Hospital of Shenzhen University. Eligible participants were individuals who had recurrent disease or who had received at least one salvage regimen without a response to standard treatment. The inclusion criteria included measurable disease, adequate physical condition, and sufficient organ function. Eligible patients (*n* = 7) were 49 to 70 years old at study entry, with an Eastern cooperative oncology group performance status score of 0–2. All patients had received multiple rounds of chemotherapy prior to CAR-T-cell therapy, and the median number of previous treatment lines was 3 (range: 2–5). One patient had previously received CAR-T-cell therapy. Five patients (71.4%) had a high/high-intermediate IPI before CD19/CD22 CAR-T-cell therapy. Furthermore, three patients (43%) had elevated LDH levels before lymphodepletion.

The primary endpoint was the ORR to treatment with CD19/CD22 BS Loop CAR-T cells, as assessed by investigators in accordance with the International Working Group (IWG) Response Criteria for Malignant Lymphoma. The ORR was defined as the percentage of patients who achieved a complete response (CR) or partial response (PR) on the basis of the IWG criteria at 1 and 3 months postinfusion. The secondary endpoint was the incidence of adverse events (AEs).

### Chimeric antigen receptor construct design

**Figure 1:**
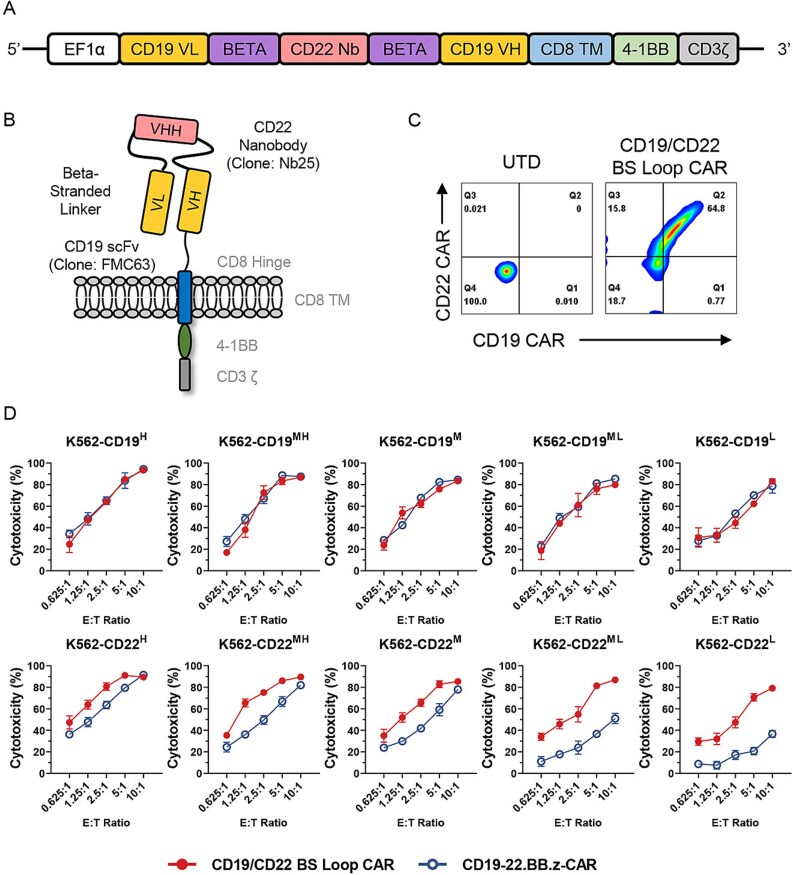
Preclinical validation of CD19/CD22 BS Loop CAR-T cells. (A) The CD19/CD22 BS Loop CAR contains the CD19 FMC63 scFv and CD22 Nb25 nanobody, a CD8 hinge and transmembrane domain, a 4-1BB costimulatory domain and a CD3ζ domain. (B) Schematic diagram of the CD19/CD22 BS Loop CAR. The binding domain of the CD19/CD22 BS Loop CAR is assembled by incorporating Nb25 between the VL and VH of FMC63 scFv via a β-stranded linker. The beta linker connecting CD19 VL and CD22 Nb utilizes the sequence EETKKYQS, while the beta linker connecting CD22 Nb and CD19 VH employs the sequence SYTYNYEK. The CD8 hinge sequence is TTTPAPRPPTPAPTIASQPLSLRPEACRPAAGGAVHTRGLDFACD. (C) Representative flow cytometry plots of the CD19/CD22 BS Loop CAR expression on primary human T cells detected by the PE-CD19 antigen and the APC-CD22 antigen. The results are representative of three independent experiments. (D) Cytotoxicity comparison of the two loop-structured CARs against different CD19- or CD22-expressing K562 variants after 24 h of incubation at different E:T ratios. The error bars represent the means ± SDs from three independent experiments. K562 variants were characterized as having a low expression level (L), a medium-low expression level (ML), a medium expression level (M), a medium-high expression level (MH) and a high expression level (H). The results from one of three independent experiments are shown.

The sequences of the bispecific CD19/CD22 BS Loop CAR (in which the anti-CD22 nanobody was incorporated between the light chain variable region (VL) and heavy chain variable region (VH) of the anti-CD19 scFv via a β-stranded linker) were obtained from the patent (CN202210126836.0). As reported previously [9], the anti-CD22 Nb25 binds the d4 domain of the extracellular domain of the CD22 antigen. The bispecific antigen-binding regions were incorporated into a second-generation CAR construct harboring the human CD8 hinge, CD8 transmembrane, 4-1BB costimulatory, and CD3ζ activation domains. A schematic of the CAR structure is provided in Fig. 1A and B.

### Manufacturing of CD19/CD22 dual-targeting CAR-T cells

As reported previously [20], for retrovirus packaging, a two-step method utilizing the sequential application of Phoenix-ECO cells followed by PG13 cells was employed to generate stable PG13 retroviral producer cell lines. Retroviruses were harvested and subsequently used for the transduction of activated human T cells. Following the acquisition of informed consent from the patients, a minimum of 2 × 10^7^ peripheral blood mononuclear cells were collected from the enrolled individuals for CAR-T-cell preparation. Fresh lymphocytes were promptly transported to a good manufacturing practice (GMP) production facility. CD3^+^ T cells were enriched via microbeads prior to activation, followed by activation with CD3/CD28 antibodies for 48 h. After activation, the T cells were subjected to genetic modification via retroviral transduction. The transfection efficiency was assessed through flow cytometry, while qPCR was employed to determine the degree of retrovirus copy number integration within each T cell. In the presence of IL-2, the enrichment process for CD19/CD22 dual-targeting CAR-T cells reached a patient-ready dose in ~12 days.

The release criteria for quality control included transduction efficiency of ≥20%, cell viability of ≥70%, amplification quantity meeting the anticipated level, and negative results from mycoplasma, bacterial, and fungal cultures. The CAR-T cells were subsequently transferred to a 0.9% sodium chloride solution supplemented with human serum albumin. The product was delivered to Union Hospital of Huazhong University of Science and Technology in Shenzhen within 2 h via a blood transport cooler maintained at 2–8°C, followed by administration to the patient under controlled conditions.

### Chimeric antigen receptor-T-cell treatment

The CAR-T cells were autologous. For the specific preparation procedures, please refer to the preceding text. All patients underwent lymphocyte depletion chemotherapy with fludarabine (30 mg/m^2^ daily for 3 days) in combination with cyclophosphamide (300 mg/m^2^ daily for 3 days) as a pretreatment regimen. CD19/CD22 CAR-T cells were infused intravenously 72 h after lymphocyte depletion. After the CAR-T-cell therapy, patient symptoms were closely monitored, and indicators such as body temperature, complete blood cell count, and coagulation function were observed regularly. The tumor status was evaluated periodically as outlined in follow-up and monitoring protocols.

### Response and toxicity assessment

The efficacy assessment adhered to the Lugano 2014 criteria and the Chinese Lymphoma Treatment Guidelines (2021 edition) [21, 22]. Imaging responses were evaluated on the basis of computed tomography/magnetic resonance imaging (CT/MRI) for a radiological response and PET/CT for a metabolic response. The treatment efficacy categories included CR, PR, SD, and PD. CRS was assessed and graded according to the American Society for Transplantation and Cellular Therapy criteria [23], whereas neurotoxicity and other AEs were evaluated on the basis of the NCI Common Terminology Criteria for Adverse Events [24], version 5.0. The focus of these studies included CAR-T-cell therapy-related CRS, ICANS, bone marrow suppression, infections, and other related complications. Adverse events were documented for all treated patients until disease relapse or death.

### Follow-up and monitoring

Following the infusion of CAR-T cells, the gene copy number of the CAR-T cells and the serum cytokine levels in the peripheral blood were monitored over a period of 3 months. The patients underwent regular follow-up assessments at 1, 3, and 6 months after infusion or until disease progression occurred. The follow-up program included physical examinations, monitoring of vital signs, laboratory tests, imaging studies, and the evaluation of treatment outcomes. The overall survival time for all patients was calculated from the date of CAR-T-cell infusion, while the progression-free survival rate was utilized to assess patient prognosis.

### Statistical analysis

For the statistical analyses conducted in preclinical studies, please refer to the corresponding figure legend. All seven patients who received infusions were included in the analysis. The measurement data are presented as medians and ranges, and comparisons were made by using the Kruskal–Wallis test. Categorical data are expressed as frequencies (%). The results were deemed statistically significant if two-sided *P* values were less than .05. The data are presented as the means ± SDs, with significance of differences determined by the Newman–Keuls multiple comparison test. All the statistical analyses were performed via GraphPad Prism version 10.1.2 software.

## Results

### Preclinical evaluation of the CD19/CD22 BS Loop CAR-T cells

Previous data have indicated that the absence of or reduction in CD19 expression serves as a mechanism of resistance following anti-CD19 CAR-T-cell therapy in patients with DLBCL [19]. As previously described [9], we engineered a CD19/CD22 bispecific CAR consisting of a single cistron encoding an anti-CD19 scFv (FMC63) and anti-CD22 nanobody (Nb25) in a loop structure (Fig. 1A and B). With retroviral vector delivery, the CD19/CD22 BS Loop CAR was consistently expressed in patient-derived T cells (Fig. 1C). To validate the sensitivity of CD19/CD22 BS Loop CAR-T cells to CD19 or CD22 antigens, the CAR-T cells were cultured with K562 cells at various antigen densities. Consistent with previous findings [9], CD19/CD22 BS Loop CAR-T cells not only demonstrated significantly enhanced cytotoxicity against CD19/CD22 double-positive tumor cell lines, surpassing that of the similar loop-structured CD19/CD22 dual-target CAR-T cells (CD19-22.BB.z-CAR) (Supplementary Fig. S1A and B), which was reported by a Stanford University group [19] but, more importantly, also exhibited significantly improved binding activity to the CD22 targets. Next, we further investigated these differences by using engineered K562 cell lines with single positivity for either CD19 or CD22. As demonstrated in our previous study [25], these K562 cell lines exhibited gradient increases in antigen density, covering a range of common wild-type leukemia and lymphoma cell lines (e.g., Raji, Daudi, IM9, and Nalm6 cells). The comparative cytotoxicity data against CD22 single-positive target cells in Fig. 1D further support this conclusion. Notably, CD19/CD22 BS Loop CAR-T cells exhibited robust cytotoxicity toward K562 cell lines with very low antigen densities (K562-CD19^L^ and K562-CD22^L^). These findings provide a rationale for advancing the clinical development of CD19/CD22 BS Loop CAR-T cells for patients with r/r DLBCL.

### CAR-T-cell product characteristics

To enhance the production consistency, quality, and cost-effectiveness of the CAR-T cell therapy product, a retroviral vector production system based on stable producer cell lines was employed in this study. Compared with transient transfection methods, this strategy significantly improves batch-to-batch consistency of viral vectors, effectively avoids residual plasmid contamination, and reduces production costs. Combined with a multi-tiered functional release testing system encompassing vector titer, transduction efficiency, and *in vitro* CAR-T cell functionality, a rigorous product quality control system was established to ensure stable biological characteristics and reproducible therapeutic efficacy. Data regarding the characteristics associated with the CD19/CD22 BS Loop CAR-T-cell infusion product are presented in Table 2. From the transduction of activated T cells to the infusion of CAR-T cells into patients, there was an average 99.4-fold expansion in CD19/CD22 BS Loop CAR-T cells and 125-fold expansion in CD19-22.BB.z-CAR-T cells. Concurrently, there was a significant enrichment in the proportion of CD4^+^ T cells during the manufacturing process (Supplementary Fig. S2A and B). The manufacturing process for CD19/CD22 BS Loop CAR-T cells was successful for all patients, with cell enrichment times of 12 to 14 days. The median transduction efficiency of the final products was 53.3% (range: 42.5%–69.2%). These findings demonstrate the potential of retroviral vectors in CAR-T-cell production, as they meet the requirements of high throughput and low cost while ensuring efficacy and safety.

**Table 2:** Characteristics summary of CD19/CD22 dual-targeted CAR-T cell drug product for all infused patients.

CAR-T therapy	Patient no.	Preparation period (days)	Cell proliferation index	Percentage of CAR-T cells	CD4/CD8 ratio of CAR-T cells
CD19/CD22 BS Loop CAR	1	12	81	53.3%	3.96
	2	14	69	45.3%	1.50
	3	13	85	42.5%	5.09
	4	13	140	58.9%	0.80
	5	12	122	69.2%	1.24
CD19-22.BB.z-CAR	6	12	77	82.4%	2.06
	7	13	173	63.9%	2.84

### Clinical trial design and patient characteristics

**Figure 2:**
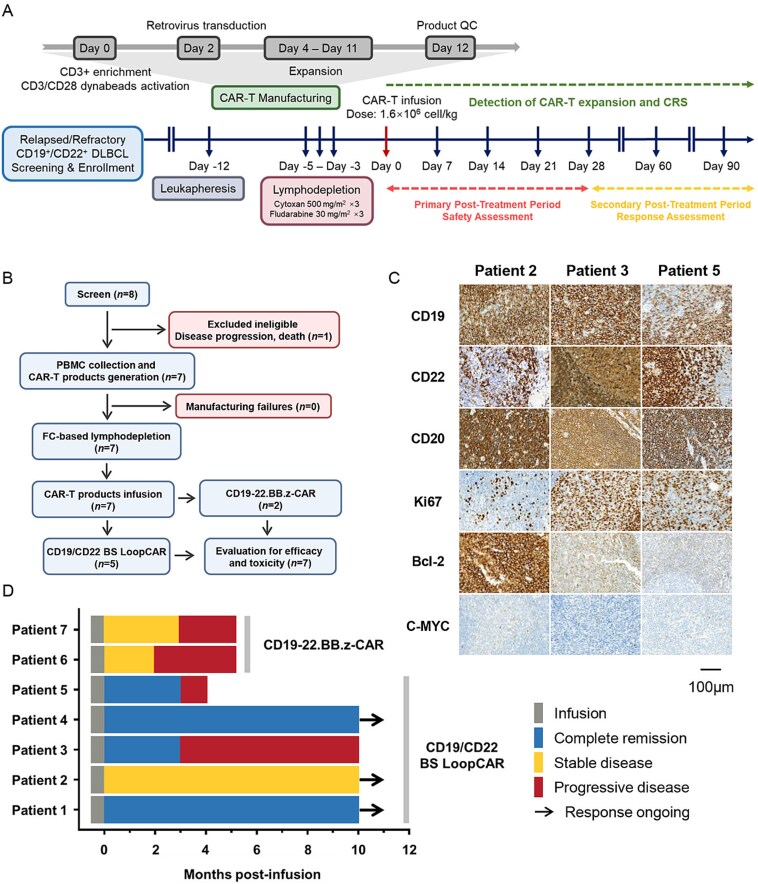
Study overview and clinical outcomes. (A) Flowchart of the CAR-T-cell manufacturing process and clinical trials. The flowchart of the clinical trial encompasses key time points, including patient screening, the manufacturing of CD19/CD22 bispecific CAR-T cells, lymphocyte depletion, CAR-T-cell infusion, and disease evaluation. After the infusion, we conducted evaluations of its safety and efficacy in the patients and collected blood samples at the arrow locations for the analysis of relevant biomarkers and CAR copy numbers. Furthermore, clinical and imaging remission evaluations were carried out at 1 month and 3 months. (B) Consort diagram of the CD19/CD22 bispecific CAR clinical trial. (C) Tissue biopsy results (CD19, CD22, CD20, Ki67, Bcl-2, and C-MYC) of three representative patients before receiving CAR-T-cell therapy. The results of the IHC assay indicated that the tumors highly expressed CD19 and CD22 antigens and that the malignant proliferation of the tumor cells was intense. (D) Swimmer plot representing the postinfusion course in each of the enrolled patients. Patients 1–5 received CD19/CD22 BS Loop CAR-T-cell therapy, and Patients 6 and 7 received CD19-22.BB.z-CAR-T-cell therapy.

From December 2023 to May 2024, we conducted a clinical trial of CD19/CD22 dual-target CAR-T-cell therapy to evaluate its efficacy and safety in patients with r/r DLBCL. After pretreatment with chemotherapeutic drugs, the patients were infused with 1.6 × 10^6^ cells/kg of the corresponding CAR-T-cell products (Fig. 2A). Efficacy and safety parameters were assessed at 1- and 3-months posttreatment, with subsequent long-term follow-up. In this clinical study of CD19/CD22 CAR-T cells, eight r/r DLBCL patients were screened for eligibility assessment, and ultimately, seven patients received treatment (two males, five females). One patient discontinued treatment because of rapid disease progression before infusion. The success rate of CAR-T-cell preparation was 100%. Five patients received a CD19/CD22 BS Loop CAR-T- cell infusion, and two patients received CD19/CD22 dual-target CAR-T cells as clinical control cases reported by Stanford University [19] (Fig. 2B). All the enrolled patients had relapsed or refractory disease after receiving ≥2 lines of prior therapy, and the coexpression of the CD19 and CD22 antigens was confirmed by Immunohistochemistry (IHC). Additional details of the patients are provided in Table 1, and the IHC results for representative patients are shown in Fig. 2C.

**Table 1:** Patient characteristics and response summary.

CAR-T therapy	Patient no.	Age/sex	Performance status (ECOG)	Disease stage (Lugano)	Prior lines of therapy	Pre-lymphodepletion LDH	IPI	CD19/CD22 expression	CAR dose (10^6^/kg)	Response (Day 30)	Maximum CRS	Neurotoxicity
CD19/CD22 BS Loop CAR	1	59/F	2	IV	2	3705	4	CD19^+^/CD22^+^	1.6	CR	I	0
	2	49/F	2	IV	5	218	4	CD19^+^/CD22^+^	1.6	SD	I	0
	3	55/M	2	I	5	165	2	CD19^+^/CD22^+^	1.6	CR	0	0
	4	68/M	1	IV	4	308	4	CD19^+^/CD22^+^	1.6	CR	0	0
	5	66/M	2	IV	2	407	4	CD19^+^/CD22^+^	1.6	CR	I	0
CD19-22.BB.z-CAR	6	70/M	0	III	2	204	2	CD19^+^/CD22^+^	1.6	SD	0	0
	7	65/M	0	IV	3	180	4	CD19^+^/CD22^+^	1.6	SD	0	0

### Clinical responses

For the assessment of the clinical response to the two CD19/CD22 CAR-T-cell therapies, a Swimmer plot was used to depict the disease response and progression in each patient (Fig. 2D). Among the patients who were treated with the CD19/CD22 BS Loop CAR-T cells, four patients (80%) achieved CR, and one patient (20%) achieved SD; the overall response rate (ORR) was 80%. Two patients maintained CR at the 3-month evaluation, and one patient remained in long-term CR during subsequent follow-ups. With the follow-up extended to May 2025 (lasting for at least 1 year), three patients experienced disease progression and eventually died, while the remaining two patients remained in complete remission. In contrast, the two patients (100%) who were treated with CD19-22.BB.z-CAR therapy achieved SD, and the ORR was 0%; both patients experienced progression within 3 months posttreatment.

**Figure 3:**
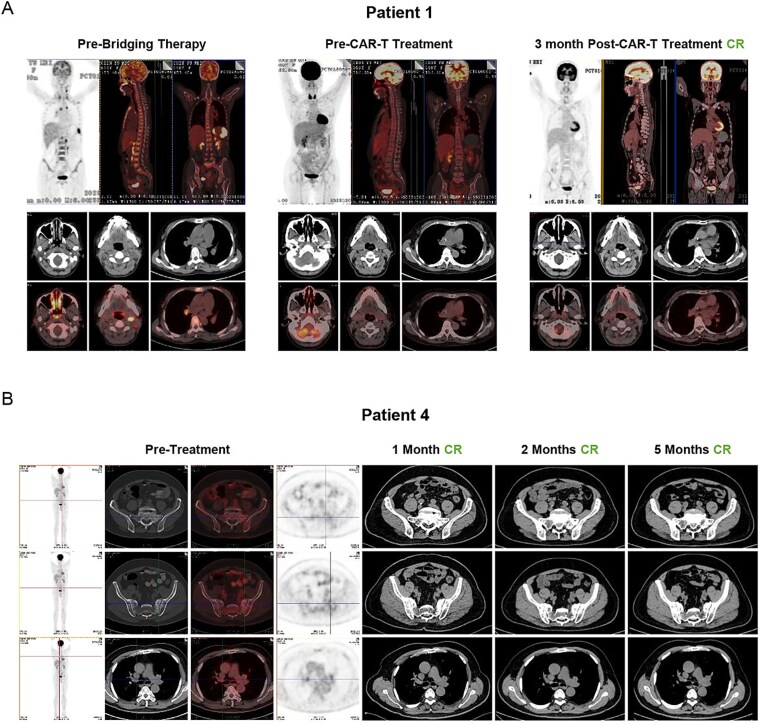
Patients in complete remission after CAR-T-cell infusion. For Patients 1 (A) and 4 (B), after the administration of CD19/CD22 BS Loop CAR-T-cell therapy, analyses of the maximum intensity projection and PET–CT composite cross-sectional imaging of the primary target lesion were conducted at the designated assessment time points. The response classifications at each time point are in accordance with the Lugano criteria.

For the patients who achieved CR after receiving CD19/CD22 BS Loop CAR-T-cell therapy, the positron emission tomography/ computedtomography (PET/CT) and CT scan results before and after treatment are shown in Fig. 3A and B. Patient 1 had r/r DLBCL of the nongerminal center B-cell-like (NonGCB) type, stage IVB, and high-risk international prognostic index (IPI). Prior to diagnosis, this patient had myelodysplastic syndrome and hemophagocytic syndrome and presented in critical condition. Before receiving CAR-T-cell therapy, the patient underwent successive HLH-1994, R-CHOP, and R-DA-EPOCH chemotherapy regimens as bridging therapies. Three months after receiving CAR-T-cell therapy, the patient tested negative for both bone marrow and peripheral blood minimal residual disease (MRD); PET/CT revealed CR. Patient 4 was successively diagnosed with DLBCL and mantle cell lymphoma (MCL) and experienced multiple relapses after undergoing several rounds of radiotherapy/chemotherapy. CT revealed a soft tissue mass near the sacrum (44 × 57 × 61 mm). The follow-up examinations at 1 and 3 months after treatment indicated MRD negativity in the bone marrow and a stable size of the soft tissue mass near the sacrum.

The number of clinical control cases was limited, and there were instances of disease recurrence. However, the overall clinical outcomes of the patient cohort suggest that CD19/CD22 BS Loop CAR-T-cell therapy offers significant clinical benefits for treating highly aggressive and lethal B-cell malignant lymphoma, particularly r/r DLBCL.

### CAR-T-cell expansion and persistence

**Figure 4:**
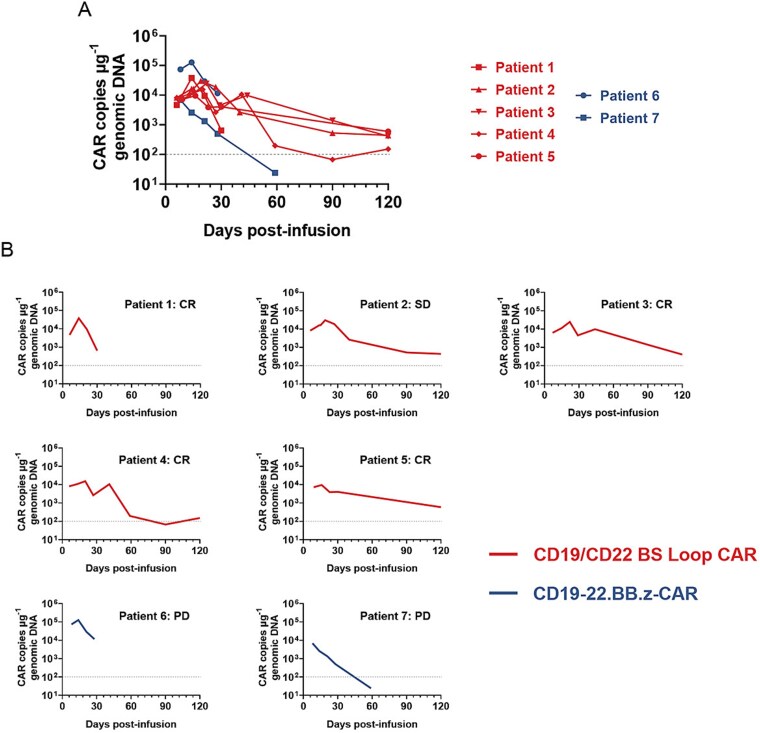
Expansion pharmacokinetics and persistence of CAR-T cells. (A) The expansion and persistence of CD19/CD22 BS Loop CAR-T cells and CD19-22.BB.z-CAR-T cells in the peripheral blood were evaluated through the detection of specific CAR sequences in genomic DNA via qPCR. The illustrations present the pharmacokinetic characteristics of CAR-T cells in the peripheral blood of all the subjects and evaluable patients at the first month and subsequent time points. Red indicates the CD19/CD22 BS Loop CAR-T-cell treatment, whereas blue represents the CD19-22.BB.z-CAR-T-cell treatment. (B) Independent display of the CAR copy number in the peripheral blood of each patient. Each graph corresponds to the result of the efficacy evaluation of the Patient 1 month after CAR-T-cell infusion. The duration of the continuous detection of CAR+ cells in the peripheral blood reached up to 4 months.

The expansion and persistence of CAR-T cells are crucial factors for assessing the efficacy of dual-targeting therapy and pose significant limitations in current research. In this study, quantitative PCR (qPCR) was utilized to detect CD19/CD22 CAR-T cells in the peripheral blood. In all evaluable patients, expansion and contraction of circulating CAR-T cells were observed, peaking at ~8–22 days after infusion (Fig. 4A and B). The median peak number of circulating CD19/CD22 BS Loop CAR-T cells, as measured by qPCR, was 24 563 (range 9593–37 663) copies of the CAR transgene per 100 ng of genomic DNA, and the median time to reach the corresponding peak level after infusion was 19 (range 14–22) days. The kinetic profiles of Patients 2, 3, and 5 shown in Fig. 4B indicate that the CAR-T gene persisted but presented a lower copy number, similar to the findings of most of the current studies. In the present analysis, only patients who experienced relapse presented undetectable CAR copy numbers, whereas all other patients presented low CAR copy numbers. Nevertheless, we could not conclude that the CAR gene copy numbers were significantly greater in patients with a good response (Supplementary Fig. S3A and B). The peak numbers of circulating CD19-22.BB.z-CAR-T cells measured by qPCR were 6846 and 126 297 copies of the CAR transgene per 100 ng of genomic DNA, respectively, and the median time to reach the corresponding peak level after infusion was 11 (range 8–14) days.

The early expansion of CD19-22.BB.z-CAR-T cells is rapid, with peak expansion occurring quickly, but the persistence in the later stage is insufficient, quickly declining to undetectable levels. This phenomenon suggests that the persistence of the CD19-22.BB.z-CAR may be slightly inferior to that of the CD19/CD22 BS Loop CAR. This finding is consistent with our previously reported preclinical data [9], as the weak response of the CD19-22.BB.z-CAR to the CD22 antigen signal leads to insufficient activation signals for T-cell expansion under repeated antigen stimulation. We therefore speculate that optimizing the single-antigen binding capacity of dual-targeting CAR-T cells is also crucial for maintaining their persistence and that this phenomenon can be further amplified in patients with highly heterogeneous antigens.

### Adverse events after CAR-T-cell infusion

**Figure 5:**
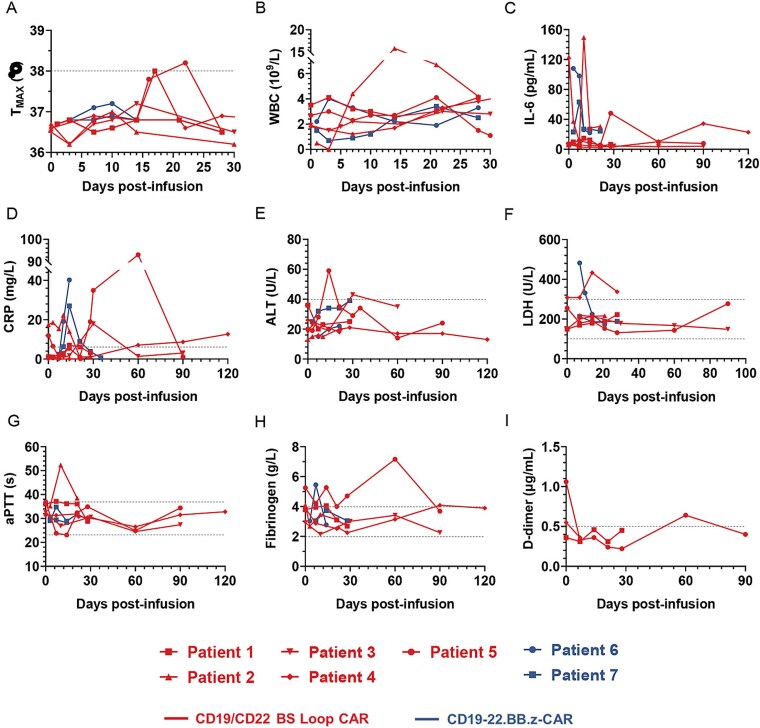
Kinetics of serum cytokines and inflammatory markers after CAR-T-cell infusion. (A and B) Changes in patient body temperature and peripheral blood WBC count after CAR-T-cell infusion. (C and D) Serum levels of cytokines correlated with the development of CRS measured at the indicated time points after cell infusion. Peripheral blood serum levels of IL-6 and CRP before and after CAR-T-cell infusion. (E–H) Serum ALT, LDH, aPTT, fibrinogen and D-dimer concentrations are shown at the indicated time points after CAR-T-cell infusion. The data of each patient are presented separately with distinct legend types. Red indicates the CD19/CD22 BS Loop CAR therapy, whereas blue represents the CD19-22.BB.z-CAR. The dotted lines indicate the boundaries of the normal values.

Tables 3 and 4 summarize the adverse events of special interest associated with the CD19/CD22 CAR-T-cell therapies, including general symptoms, hematologic toxicities, laboratory results, and findings related to the digestive, immune, and nervous systems. In patients who received CD19/CD22 BS Loop CAR-T-cell therapy, the incidence of cytokine release syndrome (CRS) was 20% (1/5). Patient 1 developed Grade I CRS 17 days after CAR-T-cell infusion and presented with a low-grade fever (maximum temperature of 38°C), which subsequently resolved following symptomatic management. The CRS duration was similar to the median duration of the CAR-T-cell expansion peak, suggesting that CRS may be due to the effective and extensive expansion of CAR-T cells. Patients receiving CD19-22.BB.z-CAR therapy did not experience CRS throughout the entire treatment process. The changes in the patient temperature and white blood cell (WBC) counts during the primary posttreatment period are detailed in Fig. 5A and B. No patients experienced CAR-T-cell-related neurotoxicity. In addition to CRS and immune effector cell-associated neurotoxicity syndrome (ICANS), we documented adverse events related to general symptoms, hematologic toxicity, laboratory abnormalities, and the digestive system. Among the five patients treated with CD19/CD22 BS Loop CAR-T cells, the main adverse reactions included fever and hematologic toxicity; three patients presented with low-grade fever, one patient developed rash, four patients had thrombocytopenia, and all five patients experienced neutropenia and developed anemia (Supplementary Table S2). Of the two patients treated with CD19-22.BB.z-CAR, one patient exhibited low-grade fever, one patient had thrombocytopenia, and both patients experienced neutropenia and developed anemia (Supplementary Table S3). All adverse symptoms were promptly alleviated with appropriate treatment.

**Table 3:** Adverse events summary for CD19/CD22 BS Loop CAR.

Adverse events	CD19/CD22 BS Loop CAR (*n* = 5)				
	**Overall** ***n* (%)**	**Grade 1** ***n* (%)**	**Grade 2** ***n* (%)**	**Grade 3** ***n* (%)**	**Grade 4** ***n* (%)**
General symptoms					
Fever	3 (60)	2 (40)	1 (20)	0	0
Shortness of breath	0	0	0	0	0
Edema of both lower limbs	0	0	0	0	0
Rash	1 (20)	1 (20)	0	0	0
Hematologic toxicities					
Decreased neutrophil count	5 (100)	0	1 (20)	1 (20)	3 (60)
Decreased platelet count	4 (80)	1 (20)	0	0	3 (60)
Anemia	5 (100)	1 (20)	1 (20)	1 (20)	2 (40)
Laboratory results					
Increased total bilirubin level	0	0	0	0	0
Increased alanine transaminase level	0	0	0	0	0
Digestive system					
Nausea/vomiting	0	0	0	0	0
Diarrhea	0	0	0	0	0
Immune system					
CRS	1 (20)	1 (20)	0	0	0
Nervous system					
ICANS	0	0	0	0	0

To evaluate the dynamic changes in inflammation and antitumor efficacy after CAR-T-cell therapy, as well as adaptive immune regulation, we measured the levels of circulating cytokines [including interleukin-2 (IL-2), IL-10, tumor necrosis factor (TNF)-α, interferons (IFN)-γ, IL-6, and C-reactive protein (CRP)] and inflammatory markers [including alanine aminotransferase (ALT), lactate dehydrogenase (LDH), WBCs, activated partial thromboplastin time (aPTT), fibrinogen, and D-dimer] at different time points. Inflammatory cytokine (IL-6 and CRP) levels demonstrated an initial rapid increase, followed by a gradual decline, indicating a broad activation of antitumor immunity, which may facilitate the subsequent robust expansion of CAR-T cells. The IL-6 level in Patient 2 significantly increased, reaching its peak at 149.4 pg/ml on Day 10 (Fig. 5C). On Day 14, the white blood cell count of Patient 2 peaked at 15.8 × 10^9^/l, which might have been associated with a transient increase in the IL-6 level. The CRP level of Patient 5 also significantly increased, to 93.05 mg/l on Day 35 (Fig. 5D). However, there was considerable variability in this parameter. Furthermore, during the monitoring period, no significant fluctuations were observed in the levels of cytokines that are associated with the efficacy and side effects (IL-2, IL-10, TNF-α, and IFN-γ), which largely remained within the normal ranges (Supplementary Fig. S4A–D). Only Patient 5 exhibited a mild elevation in IL-2 and IL-10 levels during the early treatment phase, which might have been related to the stronger initial response of the patient to CAR-T therapy (Fig. 5E and F). On the other hand, the ALT and LDH levels in all patients remained within the normal ranges during the treatment period (Fig. 5I and J). Patient 2 also exhibited coagulation dysfunction with prolonged aPTT. The remaining patients did not show any signs of coagulation dysfunction (Fig. 5K–M). Overall, regardless of the type of CAR-T cell therapy administered, the inflammatory responses and associated adverse events in the patients were effectively managed and remained within clinically controllable limits.

**Table 4:** Adverse events summary for CD19-22.BB.z-CAR.

Adverse events	CD19-22.BB.z-CAR (*n* = 2)				
	**Overall** ***n* (%)**	**Grade 1** ***n* (%)**	**Grade 2** ***n* (%)**	**Grade 3** ***n* (%)**	**Grade 4** ***n* (%)**
General symptoms					
Fever	1 (50)	1 (50)	0	0	0
Shortness of breath	0	0	0	0	0
Edema of both lower limbs	0	0	0	0	0
Rash	0	0	0	0	0
Hematologic toxicities					
Decreased neutrophil count	2 (100)	0	0	1 (50)	1 (50)
Decreased platelet count	1 (50)	1 (50)	0	0	0
Anemia	2 (100)	1 (50)	1 (50)	0	0
Laboratory results					
Increased total bilirubin level	0	0	0	0	0
Increased alanine transaminase level	0	0	0	0	0
Digestive system					
Nausea/vomiting	0	0	0	0	0
Diarrhea	0	0	0	0	0
Immune system					
CRS	0	0	0	0	0
Nervous system					
ICANS	0	0	0	0	0

### Disease progression after CAR-T-cell therapy

Patient 3, who underwent CD19/CD22 BS Loop CAR-T-cell therapy, experienced disease progression shortly after treatment. Repeat biopsy analysis revealed the presence of both CD19^+^ and CD22^+^ cells, indicating that relapse was not attributable to antigen escape. However, gene copies in the peripheral blood CAR-T cells remained detectable (Fig. 4B), suggesting that the diminished persistence of CAR-T cells was not the primary cause of relapse. Notably, this patient had received CD19 CAR-T-cell therapy 5 months prior to receiving CD19/CD22 BS Loop CAR-T-cell therapy, which initially yielded a favorable response with CR but ultimately resulted in a short duration due to insufficient CAR-T-cell persistence, leading to disease recurrence. This patient received five lines of treatment and exhibited poor responses to most chemotherapeutic agents, with tumor recurrence observed in the legs, which was indicative of significant drug resistance. On the basis of these observations, we hypothesize that a highly suppressive tumor microenvironment within this patient’s tumor hindered effective infiltration, survival, and expansion of CAR-T cells within the tumor mass. In future investigations of treatment strategies, combining targeted chemotherapeutic agents aimed at modifying the tumor microenvironment with CAR-T-cell therapy may enhance therapeutic outcomes for such patients.

## Discussion

Clinical evidence indicates that 60%–70% of r/r DLBCL patients treated with CD19 CAR-T-cell therapy either do not achieve remission or experience relapse, with ~30% of such cases attributed to antigen loss [19, 26, 27]. Single-target CAR-T cells frequently undergo downregulation or complete loss of antigen expression, whereas dual-target CAR-T cells are anticipated to mitigate this limitation [5, 8, 9, 11, 15, 19, 28, 29]. Building on our previous preclinical study [9], we have developed unique loop bispecific CAR-T cells that target CD19 and CD22 (CD19/CD22 BS Loop CAR), and we present herein the treatment outcomes for five adult patients with r/r DLBCL. The results confirmed that the CD19/CD22 BS Loop CAR not only exhibited good tolerability but also demonstrated significant clinical activity in heavily pretreated patients, with an ORR of 80%.

Despite the rapid clinical advancements associated with CD19/CD22 CAR-T-cell therapies, the rationale underlying most clinical developments and applications remains largely empirical. In designing dual-target CD19/CD22 CARs, it is crucial to consider both the stereochemical accessibility and structural characteristics of the respective target epitopes. Previous research has highlighted the importance of membrane-binding sites and hinge types for optimal CAR functionality [16–18]. For CD19 and CD22 antigens, loop-structure-based CARs have been shown to outperform tandem-based designs [18, 30]. Stanford University initiated a Phase I trial (NCT03233854) to evaluate a unique loop CD19–22 CAR in adult patients with B-cell acute lymphoblastic leukemia (B-ALL) or LBCL [19]. Within the LBCL cohort (*n* = 21), 62% of patients exhibited treatment responses, with 29% achieving CR. Relapse was correlated with the loss or diminished expression of CD19 rather than antigen escape from CD22. Laboratory investigations indicated that dual-specificity CARs elicited lower cytokine production in response to CD22, suggesting insufficient targeting efficiency for this antigen. Recruitment for the LBCL patient cohort within this clinical trial is currently suspended. Consequently, there is an urgent need to design an optimal dual-specific receptor during the development of dual-target CARs, particularly when addressing challenges related to targeting CD22.

Accurate control over the geometric structure of immune synapses is essential for optimizing CAR-T cells [9, 25]. Consequently, our objective is to maximize the cytotoxic response elicited by CD22 without compromising the signal transduction initiated by CD19, thereby preserving the conformational compatibility between CARs and dual antigen epitopes. The Nb25 that is utilized in the CD19/CD22 BS Loop CAR binds to the intermediate epitope (Domain 4) of CD22, as opposed to targeting the membrane-proximal epitope (Domains 6–7) of CD22, which is employed by the CD19-22.BB.z-CAR via M971 [31]. The incorporation of small nanobodies further facilitates the simultaneous binding of CARs to both the membrane-distal epitope of CD19 and the intermediate epitope of CD22. This design optimizes the binding distance between target cell antigens and CARs, enhancing the T-cell activation induced by CD22, which is particularly pronounced when expressed singly or at low levels on target cells. Furthermore, innovatively, in the loop CAR design, an antiparallel β-stranded linker has been introduced, demonstrating the effective display of nanobodies with compact structural domains. We believe that the 8-aa β-stranded conformation contains multiple intra- and interloop hydrogen bonds, unlike the 5-aa G_4_S linker, facilitating the correct and stable folding of the fused Nb25 and preserving its sensitivity to downregulated or diminished CD22.

The clinical data presented in this study indicate that CD19/CD22 BS Loop CAR-T cells demonstrated an 80% (4/5) ORR in r/r DLBCL patients, with four patients (80%) achieving CR and one patient (20%) achieving SD. Although this study included a small number of patients and limited data, we observed a good tolerability and encouraging therapeutic responses to the CD19/CD22 BS Loop CAR. For dual-targeting CD19/CD22 CAR therapy, Spiegel *et al.* described a safe and well-tolerated CD19/CD22 Loop CAR approach with an ORR of 62% and a CR rate of 29%; this CAR has the best bivalent loop structure reported to date and was used in our study as a reference [19]. Although two of the five patients in this study ultimately experienced relapse, all patients included in our cohort were heavily pretreated and presented with multiple refractory lesions, making this patient population particularly challenging. Consequently, we believe that this study will substantially improve the clinical management of r/r DLBCL.

Adverse events such as CRS and neurotoxicity are linked to the significant increases in serum cytokine levels following CAR-T-cell interactions with tumor cells or normal B cells, as well as the myeloid cell activation induced by CAR-T-cell engagement [32]. The primary adverse events observed in patients who received CD19/CD22 BS Loop CAR therapy included Grade I CRS (20%, 1/5), with no notable increases in serum cytokine levels and no evidence of neurotoxicity, even among patients with a substantial disease burden. In certain cases, patients exhibited reductions in platelet and white blood cell counts, bone marrow suppression or allergic rashes, which improved following treatment with anti-allergic agents, leukocyte stimulants, and platelet transfusions. Overall, the toxicity profile of the CD19/CD22 BS Loop CAR was low. Nevertheless, the sample size in this study was limited; thus, additional cases are needed to substantiate this conclusion.

While some studies have indicated that patients receiving CD19/CD22 CAR-T-cell therapy may experience CD19^−^CD22^low^ escape [5, 19, 29], such events were not observed in this study. Instead, we noted instances of antigen^+^ relapses, which have also been reported in clinical trials of CD19/CD22 CAR-T cells for B-ALL and LBCL [8, 15, 19, 29], as well as CD19/CD20 CAR-T cells for LBCL [33]. Notably, the persistence of dual-target CAR-T cells in coadministration [34] and cotransduction [35] patterns is also limited, with only a few exceptions [5, 33]. Antigen^+^ relapse is often associated with the persistence of CAR-T cells; for example, after relapse in Patients 2 and 3, the CAR gene copy numbers decreased to levels that were difficult to detect. Previous studies have demonstrated that a specific ratio of CD4:CD8 CAR to subsets (T_cm_) can increase CAR-T-cell activity [36]. Consequently, further refinements in CAR-T-cell manufacturing processes are essential to prevent premature functional dysregulation of T cells following infusion.

The tumor microenvironment in DLBCL may directly or indirectly influence CAR-T-cell functionality. Data indicate that CD8^+^ T-cell exhaustion is correlated with a poor response to axi-cel in patients with LBCL [37], and tissue biopsies have revealed high progressive disease (PD)-L1 expression in 63% of samples [38]. Retrospective analyses suggest that maintenance therapy with PD-1 inhibitors following CD19/CD22 CAR-T-cell therapy is associated with improved remission rates and survival outcomes in r/r B-Non-Hodgkin's lymphomas (B-NHL) patients, along with sustained persistence of CAR-T cells [29, 39]. We hypothesize that the combination of the CD19/CD22 BS Loop CAR with pembrolizumab may effectively address challenges such as CAR-T-cell exhaustion and immune suppression. The biopsy results revealed the presence of CD19^+^CD22^+^ cells in Patient 3 at the time of relapse, indicating significant resistance of the tumor cells and a highly suppressive tumor microenvironment. In the future, combination therapies such as Bruton’s tyrosine kinase/Phosphatidylinositol-3-kinase/B-cell lymphoma-2 inhibitors, lenalidomide, immune checkpoint inhibitors, and auto hematopoietic stem cell transplantation could be considered to further improve patient outcomes [40].

This study investigates a novel loop-structured bispecific CD19/CD22 CAR-T cell therapy. As a preliminary exploratory study, the CD19/CD22 BS Loop CAR-T cohort enrolled five patients, with primary endpoints focused on short-term efficacy and safety at 1 and 3 months postinfusion. The maximum follow-up was until May 2025 (1 year). Short-term results showed that four of the five patients achieved CR; however, with extended follow-up, two of these patients relapsed, and ultimately three patients progressed to refractory/relapsed disease and died. Further analysis of these three patients revealed common baseline characteristics including heavy pretreatment (4–5 lines), multiple prior relapses (≥2), and active uncontrolled disease before CAR-T therapy. Notably, one patient showed complete loss of both CD19 and CD22 antigen expression upon re-biopsy at relapse. Based on these findings, we speculate that tumor heterogeneity from heavy pretreatment, T-cell exhaustion [37, 38], antigen-negative escape [5, 9, 29], and uncontrolled pre-treatment disease status may collectively contribute to the failure of sustained remission.

In summary, this study demonstrates that the CD19/CD22 BS Loop CAR exhibits favorable tolerability and preliminary efficacy in patients with R/R DLBCL. Its enhanced CD22 targeting capability effectively leverages a dual-targeting approach to address antigen escape and tumor heterogeneity. However, as a preliminary exploratory analysis, this study has limitations including small sample size and relatively short follow-up period, and the current conclusions require further validation in larger studies. Based on these promising initial results, we plan to expand the clinical trial scale, extend follow-up to 2 years or longer, and broaden the scope of indications to systematically evaluate the clinical value of this unique loop-structured bispecific CAR-T cell therapy in a larger patient population, thereby providing more robust evidence for its application in treating R/R DLBCL.

## Supplementary Material

Supplementary_Materials_(Clean_Version)_tbaf027

## Data Availability

All data generated or analyzed during this study are included in this published article and its supplementary information files. Raw data are available from the corresponding author.
