# Supplementary material for: Unique loop-structured CD19/CD22 bispecific CAR-T-cell therapy for patients with relapsed/refractory diffuse large B-cell lymphoma: an observational study
Source: Antib Ther. 2025 Nov 20;9(1):24–37. doi: 10.1093/abt/tbaf027 (PMC12804173; doi:10.1093/abt/tbaf027)
Supplement: Supplementary_Materials_(Clean_Version)_tbaf027 [file supplementary_materials_(clean_version)_tbaf027.docx]

**Supplementary Materials for**

Novel Loop-Structured CD19/CD22 Bispecific CAR-T-Cell Therapy for Patients with Relapsed/Refractory Diffuse Large B-cell Lymphoma

Shuhong Li^1*^, Liqiong Liu^2*^, Zelin Liu^2*^, Jianjiang Li^1^, Huanhuan Zhou^2^, Nan Zhong^2^, Yuan Ye^2^, Lijun Zhao^3^, Xiao Liang^4^, Yuanyuan Shi^3#^, Yu J. Cao^1, 5#^, Zhi Guo^2#^

**Affiliations of Institutions:**

^1^State Key Laboratory of Chemical Oncogenomics, Shenzhen Key Laboratory of Chemical Genomics, Peking University Shenzhen Graduate School, Shenzhen, Guangdong, 518055, China

^2^Department of Hematology, Affiliated Nanshan Hospital of Shenzhen University, Shenzhen, 518052, China

^3^Shenzhen Cell Valley Biomedical Co., LTD, Shenzhen 518118, China

^4^National Engineering Research Center for Foundational Technologies for CGT Industry, Shenzhen, 518055, China

^5^Institute of Chemical Biology, Shenzhen Bay Laboratory, Shenzhen, 518132, China

***Corresponding authors:**

Zhi Guo, Tel.: +86-136-7105-3558, E-mail: guozhi77@126.com

Yu J. Cao, Tel.: +86-755-2603-3107, E-mail: [joshuacao@pku.edu.cn](mailto:joshuacao@pku.edu.cn)

Yuanyuan Shi, Tel: +86-185-6578-0299, E-mail: yshi@sz-cell.com

**This file includes:**

Methods

Figures S1 to S2

**Methods**

**Cell lines and culture conditions**

The engineered K562 cell lines were cultured in RPMI1640 complete media (Hyclone, SH30255.01) supplemented with 10% heat-inactivated fetal bovine serum (FBS) (Genstar, C511-10), 1% penicillin, 1% streptomycin, 0.1mM non-essential amino acids, 6mM L-glutamine and 1mM sodium pyruvate. CD19/CD22 bispecific CAR-T cells were cultured and expanded in X-VIVO^15^ (LONZA, 04-418Q) medium supplemented with IL-2 (Genscript, Z00368) and 5% FBS.

**Cytotoxicity assay in vitro**

CFSE-labeled tumor cells (CFSE^+^) were cocultured with CAR-T cells for 24 hours at different E:T ratios. After incubation, the dead cells were excluded by 7-AAD staining. CAR-T-cell-mediated cytotoxicity was evaluated using a flow cytometer by counting the residual live target cells (identified as 7-AAD^–^CFSE^+^). Target cell lines were a panel of stable genetically engineered cells, including different CD19- or CD22-expressing K562 cells (K562-CD19 and K562-CD22). K562 cells were transduced with the lentivirus and sorted by flow cytometry for the variants with different CD19- or CD22-expression levels as described in a previous publication (1).

**Flow cytometry**

The expression of CAR and membrane proteins was assessed using flow cytometry. Cells were pre-washed in FACS buffer (1×PBS, 3% FBS) and subsequently incubated with antibodies for 30 minutes on ice. Following two washing steps, the samples were analyzed using the BD Accuri™ C6 Plus and FlowJo software. The following antibodies were used: APC anti-human CD3, PerCP anti-human CD4, PE anti-human CD8 (all from BioLegend). For detection of CAR expression, FITC-labelled human CD19 (CD9-HF251, ACRO Biosystems) and PE-labelled human CD22 (SI2-HP2H5, ACRO Biosystems) was used.

**CAR-T** **Proliferation**

For CD19/CD22 bispecific CAR-T cells obtained by retroviral transduction, live cells were counted by trypan blue staining every 48 hours from the day of transduction, and cell growth curves were plotted.

**Immunohistochemistry**

Immunohistochemical (IHC) analysis was employed to analyze formalin-fixed and paraffin-embedded tissue sections. Briefly, after the sections were dewaxed with xylene, rehydration was carried out using a series of graded alcohols, and then endogenous peroxidases were blocked with 3% hydrogen peroxide. Antigen dissociation was performed using EDTA buffer (pH 9.0). After washing the sections in PBS, IHC staining was conducted using anti-human CD19 and anti-human CD22 antibodies. The binding of antibodies could be revealed by adding 3.30-diaminobenzidine substrate. Subsequently, the tumor tissue was further counterstained with hematoxylin.

**Assessment of CAR-T expansion and persistence *in vivo***

Following the infusion of CAR-T cells into the patient, blood samples were collected from the patient at diverse time points. Lysis buffer (BD Biosciences) was added, and genomic DNA was extracted using a genomic DNA purification kit (Thermo Fisher). A standard curve ranging from 5×10^0^ to 5×10^6^ copies/μl was constructed by tenfold dilution of the standard plasmid. The qPCR assay was conducted on the LightCycler® 480II System (Roche), and each sample was analyzed in triplicate.

**Fig. S1 Schematic diagram of structure of CD19-22.BB.z-CAR.**

**
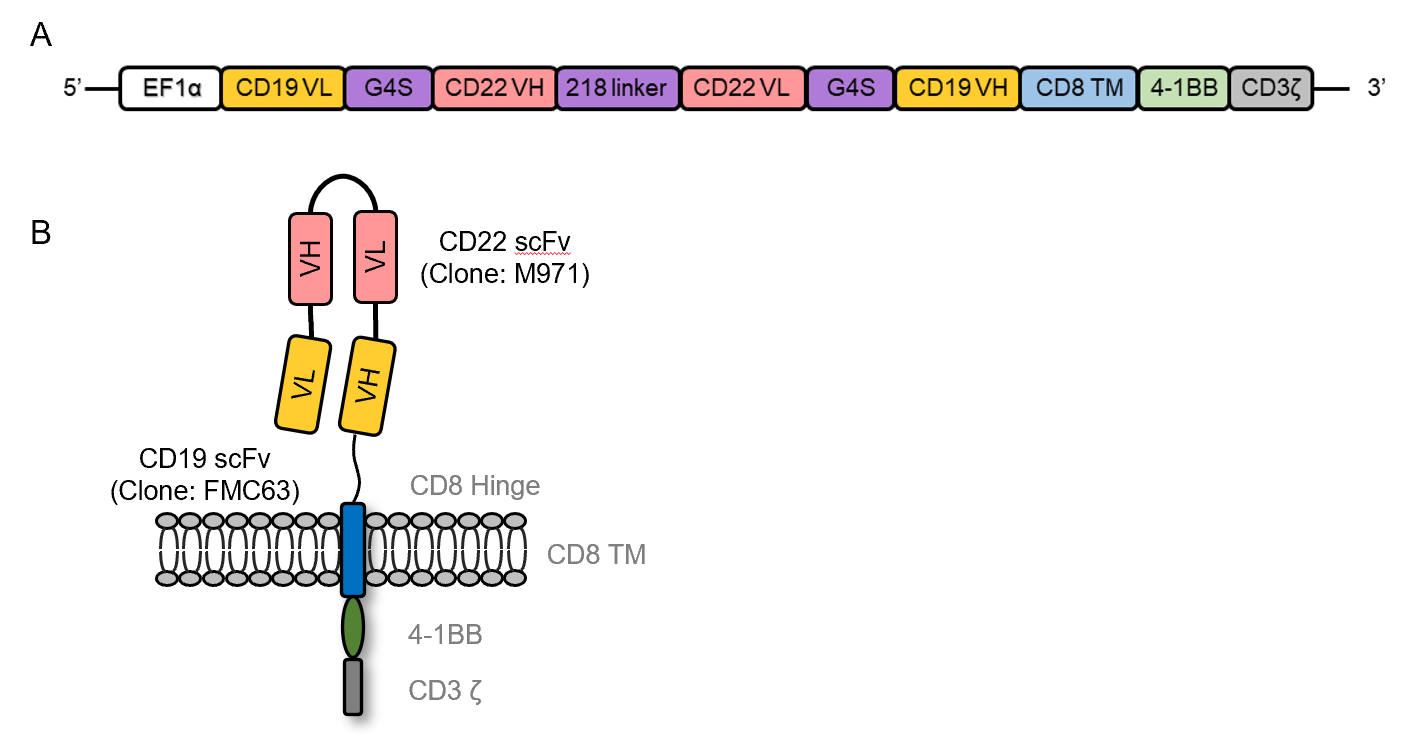
**

**Supplementary Fig. S1 Schematic diagram of structure of CD19-22.BB.z-CAR.**

**A.** The CD19-22.BB.z-CAR is composed of a targeting domain formed by inserting the M971 scFv between the VL and VH regions of the FMC63 scFv via two G4S linkers and a 218 linker, followed by a CD8 hinge and transmembrane domain, a 4-1BB costimulatory domain, and a CD3ζ domain. **B.** Schematic diagram of the CD19-22.BB.z-CAR.

**Fig. S2 Characterization of CAR products throughout the manufacturing process.**


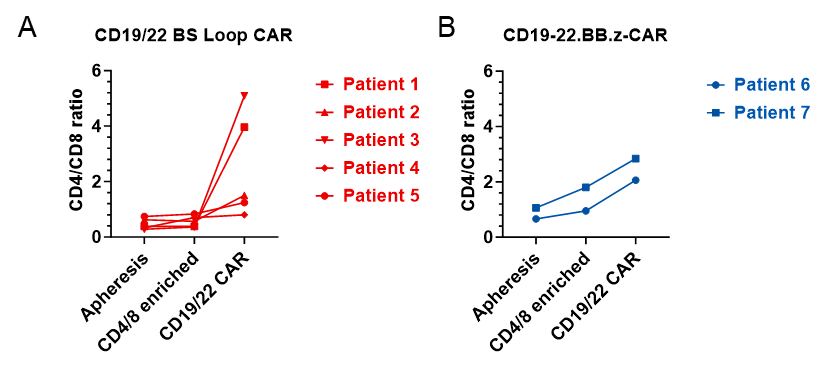


**Fig. S2 Characterization of CAR products throughout the manufacturing process.**

**A-B.** Subset composition of apheresis, CD4/8-enriched and CD19/CD22 dual-targeted CAR-T cell drug product over time. Red indicated the CD19/CD22 BS Loop CAR therapy (**A**), while blue represented the CD19-22.BB.z-CAR (**B**).

**Fig. S3 *In vivo* CAR-T cell proliferation peaks do not significantly correlate with patient treatment outcomes.**


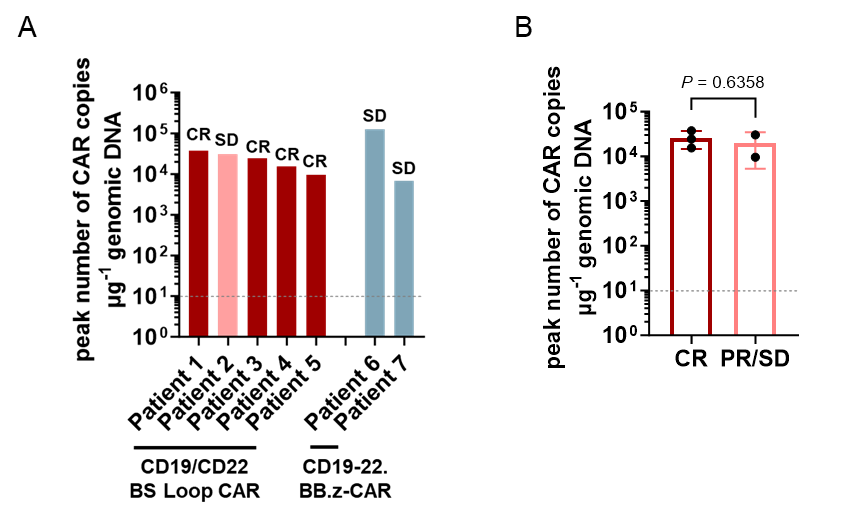


**Fig. S3 *In vivo* CAR-T cell proliferation peaks do not significantly correlate with patient treatment outcomes.**

**A-B.** Peak CD19/CD22 dual-targeted CAR-T cells as measured by qPCR compared by (**A**) product type (treatment outcomes at 1 month for each patient are marked at the top of the bars) and (**B**) 3-month treatment outcome for CD19/CD22 BS Loop CAR therapy. No significant difference observed.

**Supplementary Fig. S4 Additional data on serum cytokine kinetics after CAR-T infusion.**


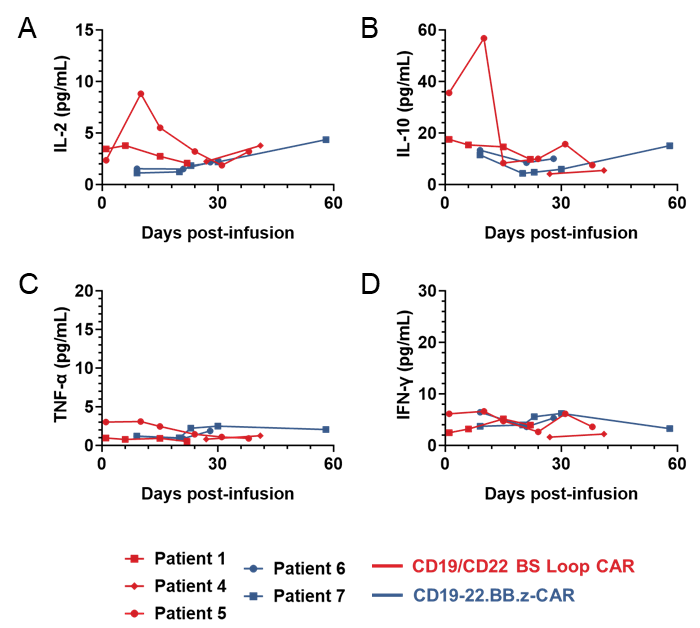


**Supplementary Fig. S4 Additional data on serum cytokine kinetics after CAR-T infusion.**

**A-D.** Kinetic of serum cytokine levels associated with efficacy and side effects during CAR-T treatment. Peripheral blood serum levels of IL-2, IL-10, TNF-α and IFN-γ, respectively. The data of each patient is presented separately with distinct legend types. Red indicated the CD19/CD22 BS Loop CAR therapy, while blue represented the CD19-22.BB.z-CAR.

**References**

1. Zhao L, Li S, Wei X, Qi X, Liu D, Liu L*, et al.* (2022) A novel CD19/CD22/CD3 trispecific antibody enhances therapeutic efficacy and overcomes immune escape against B-ALL. *Blood* 140(16):1790-1802.
